# Secondary Hemorrhage after Amputations—Comparative Advantages of Circular and Flap Amputations

**Published:** 1842-07

**Authors:** 


*Secondary hemorrhage after amputations—comparative advantages of circular and flap amputations.*—In a discussion in the Royal Medico-Chirurgical Society, May 11, 1841, "Mr. Rutherford Alcock said that the question of amputations generally, and of the different modes of performing them, and of the effects of different circumstances upon the results of the cases, had engaged much of his attention for several years. Many of the results which he had attained were already before the public, and he would now limit himself to observations on the principal topics of the present paper, and especially to the question of the comparative advantages of the flap and circular modes of operation, and the results of each with reference to the ultimate terminations of the cases, and the several accidents that supervene on amputations. With respect to the occurrence of secondary hemorrhage, he quite agreed with Mr. Liston that the oblique division of the femoral artery was not a matter of importance. But he was not sure that the oblique division of the smaller arteries had not some influence in bringing on or permitting that accident. He had often thought that, in flap amputations, the small vessels retracted only partially, which, in circular amputations, would have retracted completely; or that the vessels in the former case were overlooked, which, in the latter, bleeding distinctly, would have been secured. Nothing, however, had convinced him more of the necessity of having accurate records of series of cases, and of not trusting to general impressions, than the results of amputations. In these questions, more than in any others, general impressions were apt to be erroneous, from the circumstance of the strength of the impression which single events, deemed at the time remarkable or important, make upon the mind. With respect to this very question of secondary hemorrhage, for instance, he had himself gained a very wrong impression from two cases, which was not corrected until he had accurately ascertained the results of the whole series of those that had fallen under his observation. The two cases were these:—In an epidemic from which the troops under his charge suffered at Vittoria, the legs in several cases became gangrenous, and required amputation. In the first two instances he amputated in each patient one leg by the flap, and the other by the circular operation; and in both these cases the flap operation was followed by secondary hemorrhage; but after the circular none supervened. The circumstance made such an impression on him that he entertained no doubt that flap amputations were more obnoxious to secondary hemorrhage than circular, till he came to sum up the results of all his cases, or, at least, of all those of which he had a regular and well-kept series. The result of this examination was, that in 115 cases, of which 90 were circular amputations, and 25 flap amputations, the proportion of secondary hemorrhages was somewhat greater in the former than in the latter. In the second place, with regard to the liability to necrosis of the sawn bone in the two classes of cases, he did not think that any difference could be found between them; and it was the same with regard to the conical form of the stump, an event which he believed could only very rarely occur after either kind of amputation, if dexterously performed by a surgeon practised in the operation. As in the 115 cases alluded to it had occurred only twice, he could not draw any conclusion respecting its frequency in one or the other operation. With reference to the main question of the comparative mortalities of each mode of operation, he has found that his results were by a fraction disadvantageous to the flap amputations. He was not, therefore, prepared to agree in what was said to be the growing opinion that this operation would in time completely supersede the other. It was true that the flap amputation was a more rapid mode of operating than the circular, and to this full weight must be allowed; for pain is in itself an absorbent of life, and is always followed by a reaction and a febrile disturbance, that may have an important influence on the recovery of the patient. At the same time he thought that the difference in this respect between the two modes of operation had been overrated. Eighty or ninety seconds were ordinarily sufficient for the performance of the circular operation; and if the flap amputation were done in fifty or sixty, the difference of the times during which the patient was in each exposed to pain was really not so great that much much importance could be attributed to it. It did not appear to him, therefore, that on this ground there could be any just reason for discarding the circular mode of operation. A more important point seemed to him to be involved in the question of the after treatment of the stump. There was nothing of which he was more certain than that the idea of the advantages of union by the first intention, which was so commonly held in this country, was greatly exaggerated, and he would go so far as to say that, in some cases, such a union was not only of no advantage, but was actually mischievous, and dangerous to the life of the patient. For patients, after immediate union of the wound, would often, as he had himself seen, die of the very diseases which were said to result from the wound remaining open. In a primary amputation, after an accident in a healthy man, it was no doubt most natural to desire that the wound should be healed as quickly as possible; and, probably, in these cases, it was best to try to obtain a union by the first intention. But in secondary amputations, and in those performed for diseases of the limbs, when the incisions were made through diseased tissues, there was no use whatever, but, he believed, a disadvantage, in bringing them together. They would almost invariably open again, and place themselves in the position in which they had been left from the first. This was especially the case when limbs were amputated for diseases which had been connected with a considerable discharge. In all these it was dangerous to stop the discharge too suddenly, and it had been proposed to make an issue near the end of the stump, to keep up an artificial drain to replace that which had been cut off. But this method, he thought, would be very inconvenient, and such indeed as could scarcely be put in practice; another, which he had sometimes adopted, and which seemed to fulfil all the intention, consisted in passing a skein of silk through the lower part of the wound, and thus maintaining, for a time, a constant irritation and a discharge of matter, which afterwards might be gradually reduced."—*Am. Jour. Med. Sci.*, from *London Med. Gaz.*, May, 1841.

